# In Silico and In Vitro Investigation of the Distribution and Expression of Key Genes in the Fucose Operon of *Escherichia coli*

**DOI:** 10.3390/microorganisms11051265

**Published:** 2023-05-11

**Authors:** Nehal A. Saif, Yomna A. Hashem, Heba M. Amin, Ramy K. Aziz

**Affiliations:** 1 Department of Microbiology and Immunology, Faculty of Pharmacy, October University for Modern Sciences and Arts (MSA), Giza 12451, Egypt; 2 Department of Microbiology, Faculty of Pharmacy, The British University in Egypt, El-Sherouk City, Cairo 11837, Egypt; 3 Department of Microbiology and Immunology, Faculty of Pharmacy, Cairo University, Cairo 11562, Egypt; 4 Center for Genome and Microbiome Research, Cairo University, Cairo 11562, Egypt; 5 Microbiology and Immunology Research Program, Children’s Cancer Hospital Egypt 57357, Cairo 11617, Egypt

**Keywords:** *E. coli*, fucose, comparative genomic analysis, real-time PCR, subsystem, SEED

## Abstract

Many gut bacteria degrade polysaccharides, providing nutritional advantages to their hosts. Fucose, a mucin degradation product, was suggested as a communication molecule between the resident microbiota and external pathogens. However, the precise role and variants of the fucose utilization pathway remain to be elucidated. Here, we computationally and experimentally investigated the fucose utilization operon of *E. coli*. While the operon is conserved among *E. coli* genomes, a variant pathway, in which an ABC transporter system replaces the fucose permease gene (*fucP*), was computationally identified in 50 out of 1058 genomes. Comparative genomics and subsystems analysis results were confirmed by polymerase chain reaction-based screening of 40 human *E. coli* isolates, which indicated the conservation of *fucP* in 92.5% of the isolates (vs. 7.5% of its suggested alternative, *yjfF*). The in silico predictions were confirmed by in vitro experiments comparing the growth of *E. coli* strains K12, BL21, and isogenic fucose-utilization K12 mutants. Additionally, *fucP* and *fucI* transcripts were quantified in *E. coli* K12 and BL21, after in silico analysis of their expression in 483 public transcriptomes. In conclusion, *E. coli* utilizes fucose by two pathway variants, with measurable transcriptional differences. Future studies will explore this variation’s impact on signaling and virulence.

## 1. Introduction

The gastrointestinal tract (GIT) is inhabited by trillions of commensal microorganisms, which play crucial roles in human physiology, constituting the human microbiota. The fundamental relationship between the microbiota and its host relies on chemical signaling and nutrient availability. Invading pathogens compete for these resources by precisely coordinating virulence traits [1].

The GIT is lined by a protective mucus barrier, the key ingredients of which are mucins, which are large heavily glycosylated proteins that can contain up to 80% carbohydrates by weight. They are the key ingredients of the mucus barrier in GIT [2]. Seven different core oligosaccharides have been identified in human mucin; these are typically decorated with additional N-acetyllactosamine residues and then capped with sialic acid or fucose [3].

Various bacterial members of the normal microbiota, e.g., *Bacteroides thetaiotaomicron*, encode glycoside hydrolases, particularly sialidases, and fucosidases, which can remove these sugar residues from mucin. Since these hydrolases are typically secreted or occur as cell surface-associated enzymes, the released monosaccharides are available for uptake by the hydrolase-producing organism as well as other bacteria in the vicinity [3].

Mucin degradation can benefit GI pathogens in several ways. The first and simplest way is that microbiota-liberated glycans can be used as a carbon source to fuel the growth of pathogens in the lumen of the gut. Mucin O-glycan hydrolases are highly expressed during growth in the ceca of mice fed a polysaccharide-poor diet. Furthermore, mutation of five of these polysaccharide utilization loci compromises *Bacteroides thetaiotaomicron*’s ability to colonize the gut of adult mice fed a polysaccharide-poor diet, underlining the importance of mucin O-glycans as a carbon source in the absence of dietary glycans [4]. Other gut bacterial species, e.g., *Bifidobacterium bifidum*, are known to degrade mucin O-glycans [5]. Microbiota-liberated mucin O-glycans can also act as a chemical cue to help pathogens sense their surroundings in vivo [6].

Commensal *E. coli* are assumed to use a variety of mucus-derived metabolites [7], such as sialic acid, N-acetylglucosamine, gluconate, and N-acetylneuraminic acid, in addition to arabinose and fucose—the focus metabolite of this study.

Fucose is a naturally occurring deoxyhexose sugar found in the glycans on the surfaces of mammalian and bacterial cells and in the membranes and cell walls of many plants and insect species. In *Escherichia coli* and presumably *Salmonella enterica*, fucose is metabolized via parallel pathways that converge at the production of lactaldehyde and dihydroxyacetone phosphate (DHAP). DHAP can then feed into the glycolytic pathway where it can be converted to a variety of fermentation products, whereas lactaldehyde is converted to 1, 2-propanediol (PDO) and secreted [8].

In *S. enterica*, 1, 2-PDO can be taken back up and converted to propanol and propionate by enzymes end-coded by the *pdu* microcompartment locus [8]. In *E. coli*, L-fucose is utilized as a sole carbon and energy source through parallel pathways. For the uptake of L-fucose, *E. coli* uses the *fuc*-regulon consisting of a permease, an isomerase, a kinase, an aldolase, an oxidoreductase, and a regulatory protein [9].

Under aerobic conditions, the aldehyde is irreversibly oxidized to L-lactate by a NAD-linked dehydrogenase, which is encoded by *aldA*. L-lactate then enters the general metabolic pool as it gets converted to pyruvate. The pathway is different under anaerobic conditions, as the aldehyde is reduced to L-1, 2-PDO by an NADH-linked oxidoreductase encoded by *fucO*. PDO is then excreted into the medium as a terminal fermentation product. Most *E. coli* strains are unable to grow on 1, 2-PDO. Yet, when adapted to grow on 1, 2-PDO, *E. coli* does so through AldA [10], which has also been suggested as a candidate drug target, when co-targeted with PrpC [11].

During aerobic growth, the cell minimizes the reduction of lactaldehyde by synthesizing PDO oxidoreductase (or modifying it post-translationally) as molecules with low enzymatic activity or as molecules mostly in a form without catalytic activity. During anaerobic growth, the oxidation of lactaldehyde is prevented by the absence of aldehyde dehydrogenase activity, as well as the lack of necessary electron acceptors [7].

In recent years, a large body of literature provided evidence that enteropathogens are equipped with a large set of specific metabolic pathways to overcome nutritional limitations in vivo, thus increasing bacterial fitness during infection. These adaptations include the degradation of myoinositol, ethanolamine cleaved from phospholipids, fucose derived from mucosal glycoconjugates, 1, 2-PDO as the fermentation product of fucose or rhamnose, and several other metabolites not accessible for commensal bacteria or present in competition-free microenvironments [7,10,12,13].

As *E. coli* is a typical member of the gut microbiota, it is interesting to investigate whether pathogenic *E. coli* strains differ from commensal strains with respect to carbon and energy sources. The goal of this study is to investigate the distribution and expression of fucose utilization genes/operons in different *E. coli* strains.

## 2. Materials and Methods

### 2.1. Microorganisms

The bacterial strains used in this study are *E. coli* K12-MG1655, *E. coli* BL21, and mutant strains. Isogenic bacterial strains were obtained from the Keio collection, which is a collection consisting of 3985 deletion derivatives of *E. coli* K-12 strain BW25113 [14].

For genetic screening, 60 clinical bacterial isolates were obtained from biobanked stocks in the clinical laboratories of Qasr El-Ainy Educational Hospital, Abu-El-Rish Children Hospital, and the National Cancer Institute in Cairo, Egypt, in the period between February and May 2016. All isolates were screened by conventional microbiological techniques (microscopy, biochemistry, and culture methods), and 40 were identified and confirmed to be *E. coli*.

### 2.2. Phenotypic Detection of Fucose Utilization

#### 2.2.1. Carbohydrate Degradation by *E. coli* Wild-Type and Mutant Strains

*E. coli* strains and mutants were grown on MacConkey agar plates supplemented with 25 mM fucose, and cultures were observed for acidification (pink color production). For 5–10 mL starter cultures of each *E. coli* K12, BL21, and mutant strains, aliquots from glycerol stocks were grown overnight at 37 °C, with constant shaking at 250 rpm, in M9 minimal medium supplemented with proper carbon source. The overnight culture was centrifuged at 1000× *g* at 4 °C for 5 min. The pellets were washed twice with phosphate-buffered saline (PBS) solution and then gently resuspended in 10 mL of M9 minimal medium [15]. After the concentrations were calculated to reach a starting optical density at 600 nm (OD 600) of 0.05, 200–300 μL of the culture was added to a fresh medium in a total volume of 10 mL, at each tested condition. The OD was measured at 600 nm and normalized to a starting OD600 of 0.05; highly reproducible results with consistent lag times were obtained. Unless otherwise noted, at least three independent experiments were carried out for all strains in all tested growth conditions. In comparing growth patterns, glucose, which has been a more suitable fermentation substrate for *E. coli* [16], was used as control.

The composition of the M9 medium is well documented, and we used it exactly as described by Fong et al. [17]. The media contains essential salts, buffers, EDTA, and trace elements, which were prepared as: FeCl_3_·6H_2_O (16.67 g L^−1^), ZnSO_4_·7H_2_O (0.18 g L^−1^), CuCl_2_·2H_2_O (0.12 g L^−1^), MnSO_4_·H_2_O (0.12 g L^−1^), and CoCl_2_·6H_2_O (0.18 g L^−1^).

#### 2.2.2. Molecular Identification of Fucose Utilization Genes by the Polymerase Chain Reaction

The polymerase chain reaction (PCR) was used to detect the presence of fucose utilization genes in *E. coli*, as previously described [18]. Bacterial colonies were boiled in TE buffer for 5 min, flash centrifuged, and then the DNA-containing supernatant was used in the amplification reactions. MyTaq Master Mix (Bioline, London, UK) was used for all amplifications. The primers used in this study are shown in Table 1. Amplification reactions started with an initial denaturation at 94 °C for 5 min; 30 cycles of denaturation at 94 °C for 1 min, annealing at 55 °C for 45 s, and elongation at 72 °C for 1 min; then a final elongation step at 72 °C for 10 min [15].

#### 2.2.3. Nucleotide Sequence of Amplified Fucose Utilization Genes

To confirm the identity of fucose utilization genes detected by PCR, and to determine any possible new genetic variants specific to this study, we extracted the DNA from the gel bands representing positive PCR products using QIAGen PCR purification kit (QIAGen, Germantown, MD, USA). This DNA was afterward sequenced by the Sanger chain termination method at Macrogen, Korea, with the primers used for amplification. All obtained sequences were visually revised and corrected for any electropherogram ambiguities, then were compared to nucleotide and protein sequence databases by BlastN and BlastX [19], respectively, with default settings and parameters of the NCBI Blast platform (URL: https://blast.ncbi.nlm.nih.gov, accessed on 1 July 2022).

### 2.3. Subsystems Annotation Using SEED Database

The comparative genomic analysis was conducted by the subsystems approach [20] on the SEED server [21]. The SEED holds thousands of genomes and provides consistent and accurate annotations for these genomes. It is also a platform that can enable the discovery of de novo annotations [22]. The database relies on the subsystem approach; a subsystem is a set of functional roles that a user can connect to specific genes in specific genomes producing a subsystem spreadsheet [20]. This approach greatly facilitates the interpretation of genomes and pathways and can be applied to understanding cellular networks, gene and pathway discovery, identification of novel drug targets, and strain engineering [22].

The subsystem technology was used in this study to curate a subsystem entitled “L-fucose utilization and AldA biosynthesis” on the SEED database, which was an expansion of a previously set in the subsystem (L-fucose utilization), curated by Dimitry Rodionov [23]. The subsystem included the fucose regulon genes, consisting of a permease, the isomerase, a kinase, an aldolase, an oxidoreductase, and a regulatory protein.

### 2.4. Comparative Genomic Analysis

The SEED database [24] and the analysis tools wherein were used for operon analysis, subsystem construction, and editing. Artemis was used for whole genome analysis, editing, and visualization [25]. The KEGG database (URL: https://www.genome.jp/kegg/kegg2.html, last accessed on 1 January 2022) was used for pathway visualization [26] along with the transport classification database (URL: http://www.tcdb.org/, accessed on 1 March 2020), which was used for classification of membrane transport proteins [27].

The fucose operon was analyzed in a set of genomes of the family Enterobacteriaceae, including *E. coli*, *Salmonella*, *Shigella*, and *Citrobacter*. Comparative analysis to predict different genes responsible for fucose utilization in these selected species was performed in the SEED database through the selection of genomes from these organisms. All genes were compared in genomes of the above organisms to find variable patterns in fucose utilization, with the reference organism arbitrarily set to *E. coli* strain K12 substrain MG1655. STRING (URL: https://string-db.org, accessed on 1 March 2022), an in silico platform for functional protein association networks [28], was used to determine the proteins transcribed by the genes in the study.

### 2.5. In Silico Gene Expression Analysis

The Gene Expression Omnibus (GEO) portal (URL: http://www.ncbi.nlm.nih.gov/geo/ (accessed on 1 June 2020) was used to search publicly available datasets for recent studies of *E. coli* RNA analysis with at least three samples representing all fucose operon genes and performed by whole-genome microarray chips [29]. The microarray expression data of 483 samples in 52 datasets (Supplementary Materials Table S1) were analyzed in the R statistical package (downloaded from URL: https://www.r-project.org on 1 August 2018) and visualized by RStudio (R version 3.4.4/R Studio version 1.1.442) [30].

### 2.6. RNA Extraction

For RNA extraction, bacterial cells were grown overnight in M9-minimal medium containing 0.4% glucose and 0.4% fucose. The overnight samples were diluted at 1:50 to reach OD 600 of 0.05 and then subcultured in fresh media to OD 600 of 0.5–0.7. A total of 1 mL of culture was then transferred to a 1.5 mL microcentrifuge tube and centrifuged for 2 min at maximum speed. The supernatant was removed, and the pellet was washed twice with PBS to ensure the complete removal of any traces of the supernatant.

The RNeasy Mini Kit (Qiagen, Hilden, Germany) was used, according to the manufacturer’s instructions, to extract the total bacterial RNA, which was immediately treated with DNaseI for the removal of any traces of genomic DNA. RNA concentration and quality were determined by a nanophotometric device (Thermofisher Scientific, Waltham, MA, USA).

### 2.7. Reverse Transcription and Quantitative Real-Time PCR (qRT-PCR)

Quantitative real-time reverse transcription PCR (qRT-PCR) was used to study the expression of different fucose utilization genes in different *E. coli* spp. after a reverse transcription step.

For reverse transcription, the SensiFAST^TM^ cDNA synthesis kit (Bioline, UK, Catalog number IO-65053) was used to generate cDNA from 500 ng to 1 μg of DNase-treated RNA. The reaction components were mixed and kept on ice. The mixture was added in a thermal cycler with a program of: primer annealing at 25 °C for 10 min, reverse transcription at 42 °C for 15 min, and finally inactivation at 85 °C for 5 min. The reaction was maintained at 4 °C in a thermal cycler till stored at −20 °C till use.

For real-time PCR, the Quantitect SYBR green master mix (Qiagen, Hilden, Germany) was used to amplify and quantify the cDNA. The *ihfB* gene [31] was used as a housekeeping gene to normalize the results obtained with all analyzed genes in this study. The real-time reaction was carried out in StepOnePlus Real-Time PCR System (Thermofisher Scientific, Waltham, MA, USA), following the reaction profile instructions supplied with the QuantiTect SYBR green master mix. A melting curve was performed for each gene to confirm that the amplicon is a single product. Data analysis, applying the delta-delta cycle threshold (ΔΔCt) relative quantification method, was performed to determine the relative RNA transcripts for each gene upon growth in a minimal medium supplemented with fucose. Growth in 0.4% glucose was used as the calibrator condition.

### 2.8. Statistical Analysis

For two-variable comparisons, Student’s *t*-test was used to determine the significance of the differences between means. For multiple variable comparisons, analysis of variance (ANOVA) was used with post hoc pairwise tests. Statistical tests were either performed in the R environment (URL: https://www.r-project.org, downloaded on 1 August 2018)) and visualized by RStudio (R version 3.4.4/R Studio, version 1.1.442) [30], or analyzed and visualized by GraphPad Prism (academic copy to Cairo University, by GraphPad Software, San Diego, CA, USA).

## 3. Results

### 3.1. Comparative Genomics and Subsystems Analysis Uncover Pathway Variants of Fucose Utilization among Different Members of Enterobacteriaceae

For comparative genomics among selected members of Enterobacteriaceae, a subsystem entitled “L-fucose utilization and aldA biosynthesis” was constructed and is publicly available (URL: http://pubseed.theseed.org//SubsysEditor.cgi?page=ShowSubsystem&subsystem=L-fucose_utilization_and_AldA_biosynthesis, last accessed on 1 March 2022). Analysis of this subsystem indicated different fucose pathway variants, some of which are characterized by a missing *fucP* (Table 2). The analysis was performed on 1058 *E. coli* genomes, 373 *Salmonella* genomes, 18 *Shigella* genomes, and 15 *Citrobacter* genomes.

**Figure 1:**
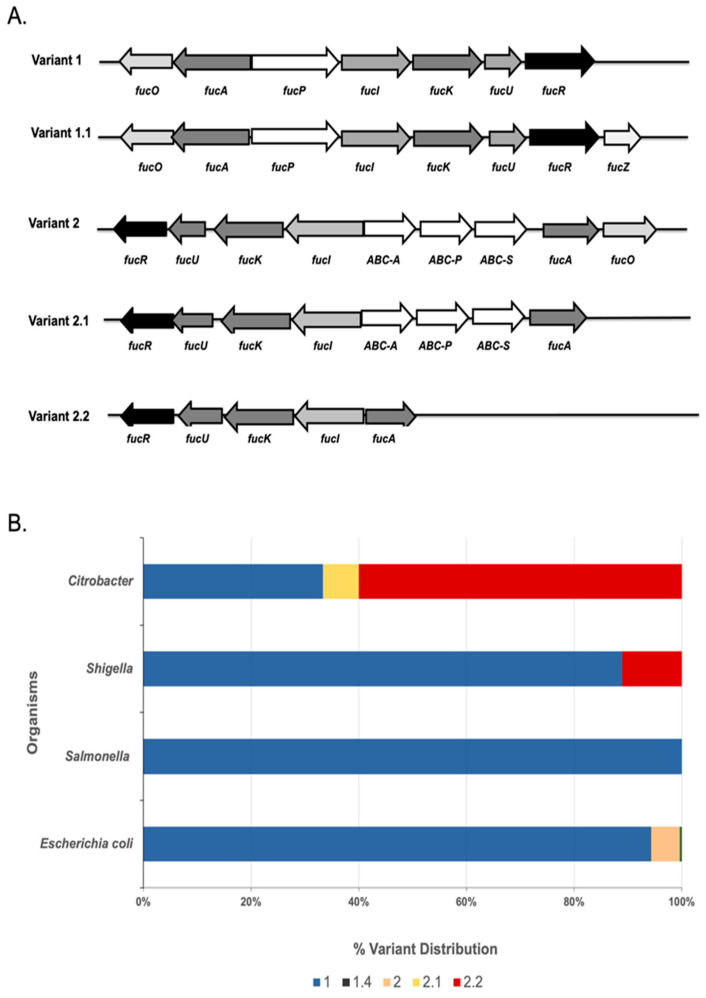
(**A**). Genetic maps of fucose operon variants among *Enterobacteriaceae*. (**B**). Variant distribution of the fucose operon in various enterobacterial species.

**Figure 2:**
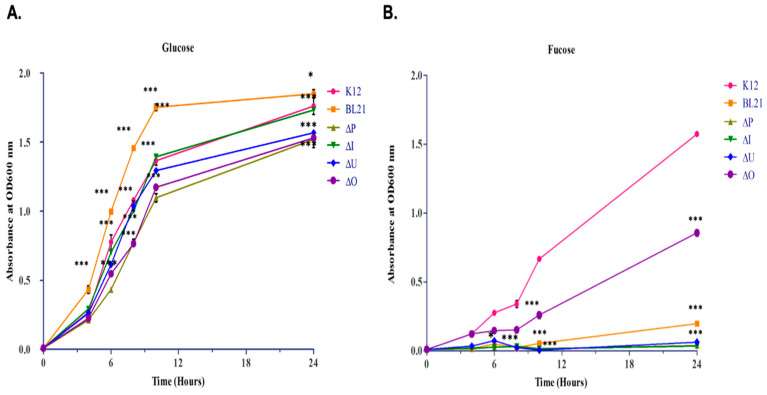
Growth curves of different *E. coli* strains incubated in minimal medium supplemented with 0.4% *w*/*v* glucose (**A**) or 0.4% *w*/*v* fucose (**B**) as the sole carbon source. The X-axis represents different time intervals: 0, 2, 4, 6, 8, 10, and 24 h and the Y-axis represents OD600 of samples. The data were analyzed for statistical significance by ANOVA, followed by post hoc pairwise analysis with Bonferroni correction. Significant differences from the wild-type K12 strain are indicated by * (0.01 ≤ *p* < 0.05) r *** (*p* < 0.001). Full pairwise comparisons are provided as Supplementary Material (Supplementary Table S2).

The constructed subsystem highlighted four major variants of the fucose utilization operon among Enterobacteriaceae (Table 2). *E. coli* genomes have two major variants (1 and 2) and two subvariants (1.1 and 2.1. Figure 1A). The majority of analyzed *E. coli* strains (86%) have a Variant 1 structure (Figure 2B), which is typically the most described fucose operon pathway [32]. Among the representatives of Variant 1 is *E. coli* strains K12, O104:H4, and O157:H7, while a representative of Variant 2 is BL21. The key difference between variants 1 and 2 is in the presence or absence of the FucP permease transporter-encoding gene, respectively. In Variant 2, a gene encoding a putative ABC-type transporter sometimes replaces *fucP*. According to STRING analysis, the nucleotide sequence of yjfF encodes a protein that is part of the ABC transporter complex. Thus, this gene is presumably responsible for the translocation of the substrate across the membrane in strains lacking *fucP*, such as BL21.

*Shigella* is similar to *E. coli* in its fucose operon structure, with a slight difference in that some strains have a structure belonging to Variant 2.2 (11%). In *Citrobacter*, however, 60% of the strains have the Variant 2.2 structure, with no ABC transporter or permease genes. *Salmonella* has only two different variants related to the fucose utilization pathway: Variant 1 (89.7%) and Variant 1.1 (10.3%) (Figure 1B).

### 3.2. In Vitro Screening of Genetic Determinants Involved in Fucose Sugar Utilization

To test whether the genetic variation observed among the publicly available E. coli genomes is reflected in locally isolated specimens, we screened 40 clinical isolates, phenotypically confirmed as *E. coli* (Supplementary Materials Figure S1), for the presence of genes that represent the two fucose operon variants 1 and 2. Of note, the comparative genomics analysis highlighted *fucP* as the major differentiating gene between the two variants. Thus, *fucP* and *fucI* were screened by PCR followed by gel-electrophoresis of PCR-amplified products, and they were detected in 92.5% (37/40) and 95% (38/40) of the samples, respectively. The *yjfF* gene, representing the ABC transporter that was computationally predicted to substitute *fucP*, was screened by PCR and was found in the three samples (7.5%) that lacked the *fucP* gene. A representative of each PCR product was confirmed by sequencing to ensure specificity.

### 3.3. Differential Utilization of Glucose and Fucose by Wild-Type and Mutant E. coli Strains

After molecular (genotypic) validation of predicted pathway variants, we set out to validate the phenotypic difference between them. To confirm that *E. coli* K12 could utilize fucose, as predicted from its genome, we cultured the bacterial strain in the M9 chemically defined medium, supplemented with either fucose or glucose (0.4% *w*/*v*) as the sole carbon source, while growth on glucose was monitored for comparison. *E. coli* K12 could use each of the two monosaccharides as the sole carbon source (Figure 2); however, differences in growth patterns were observed when K12 was compared with BL21 and different mutant strains from the Keio collection (Figure 2).

Incubation of K12 with glucose resulted in faster growth and thus a higher cell yield during the first 10 h than with fucose (*p* < 0.001 Figure 2A vs. Figure 2B); but after 24 h, incubation with fucose resulted in comparable, albeit slightly lower, final optical density (Figure 2B). BL21 grew faster on glucose than K12 did, with a significantly higher cell yield (*p* < 0.001 Figure 2A). On the other hand, incubation of BL21 with fucose resulted in lower growth efficiency than K12 (extended lag phase, lower growth rate, and lower biomass, Figure 2B). Growth of Δ*fucP* mutant on M9 minimal medium, supplemented with fucose, showed the same significant pattern as BL21 (*p* < 0.001). However, Δ*fucI* failed to grow on the minimal medium supplemented with fucose. The growth pattern of Δ*fucU* was characterized by an extended lag phase. Δ*fucO* shared a similar pattern to the growth of K12 on minimal media supplemented with fucose (Figure 2B).

In addition to K12 and BL21, all clinical *E. coli* isolates used in the study were confirmed to ferment fucose by growth on MacConkey agar plates supplemented with fucose instead of lactose.

### 3.4. In Silico Meta-Analysis of Gene Expression Data Reveals the High Expression of fucP in Selected Sugars and Growth Phase

Normalized microarray expression data from 483 samples in 52 datasets collected from GEO were plotted against different conditions. The expression of each gene was analyzed at different growth phases, in different culture media, and in the presence of different carbon sources.

**Figure 3:**
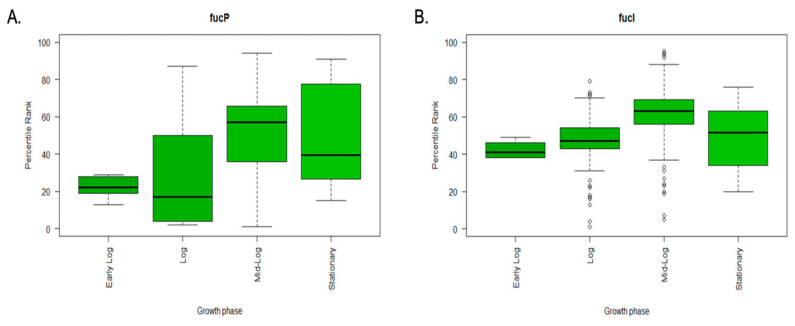
Boxplots showing the differential expression of different genes at different growth conditions. Percentile rank expression data were retrieved from the GEO database, tabulated, and plotted in R Studio. (**A**). Boxplots showing the differential expression of *fucP* at different growth phases. (**B**). Boxplots showing the differential expression of *fucI* at different growth phases.

**Figure 4:**
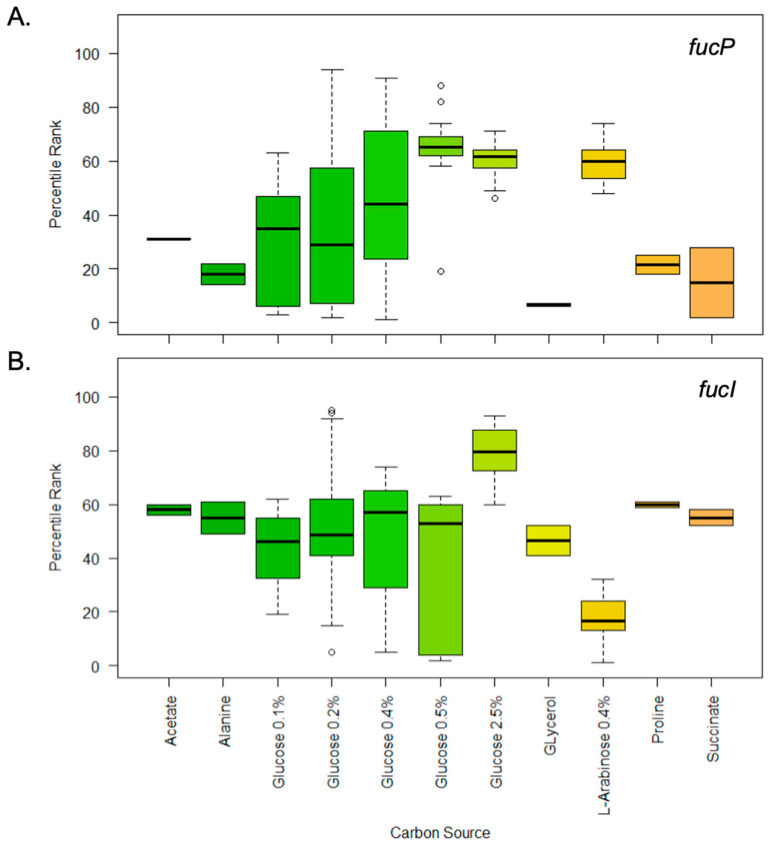
Boxplots showing the differential expression of *fucP* and *fucI* under different carbon sources. Percentile rank expression data were retrieved from the GEO database, tabulated, and plotted in R Studio. (**A**). Boxplots showing the differential expression of *fucP* when the bacteria were grown in different carbon sources. (**B**). Boxplots showing the differential expression of *fucI* when the bacteria were grown in different carbon sources. Carbon sources are shown at the bottom, for both panels (**A**,**B**).

This analysis showed that the *fucP* gene was most highly expressed in the mid-log phase (highest median in Figure 3A) and upon growth in media supplemented with 0.2% glucose. However, the expression was extremely low in the early log phase and upon culturing in media supplemented with 0.1% glucose, or supplemented with 30 mM glucose and 0.4% L-arabinose (Figure 4A).

Similarly, the *fucI* gene was highly expressed in the mid-log phase (Figure 3B) and upon growth in media supplemented with 0.2% glucose (Figure 4B). Like *fucP*, *fucI* seemed to have its highest expression at the mid-log phase; however, it had higher growth at the log phase (compare medians in Figure 3B vs. Figure 3A). It also differed when it came to carbon source, and the expression was extremely low upon culturing in minimal medium, as will be shown later.

### 3.5. In Vitro Expression Analysis of Fucose Utilization Genes in M9 Minimal Medium Supplemented with Glucose and Fucose

After predictions were made about the optimal conditions for expression of the two selected fucose operon genes, *fucI* and *fucP*, the transcript levels of these genes were estimated in minimal media supplemented with glucose and fucose. First, the expression level of the fucose utilization genes, *fucP*, and *fucI*, under the effect of fucose was investigated by real-time RT PCR. The amplification profile of the housekeeping *ihfB* gene in both runs (with glucose and fucose) showed quite close cycle numbers indicating that RNA quantities used from the two conditions are relatively close. At the same time, the amplification profiles of *fucP* and *fucI* under treated and untreated conditions had different Ct values.

**Figure 5:**
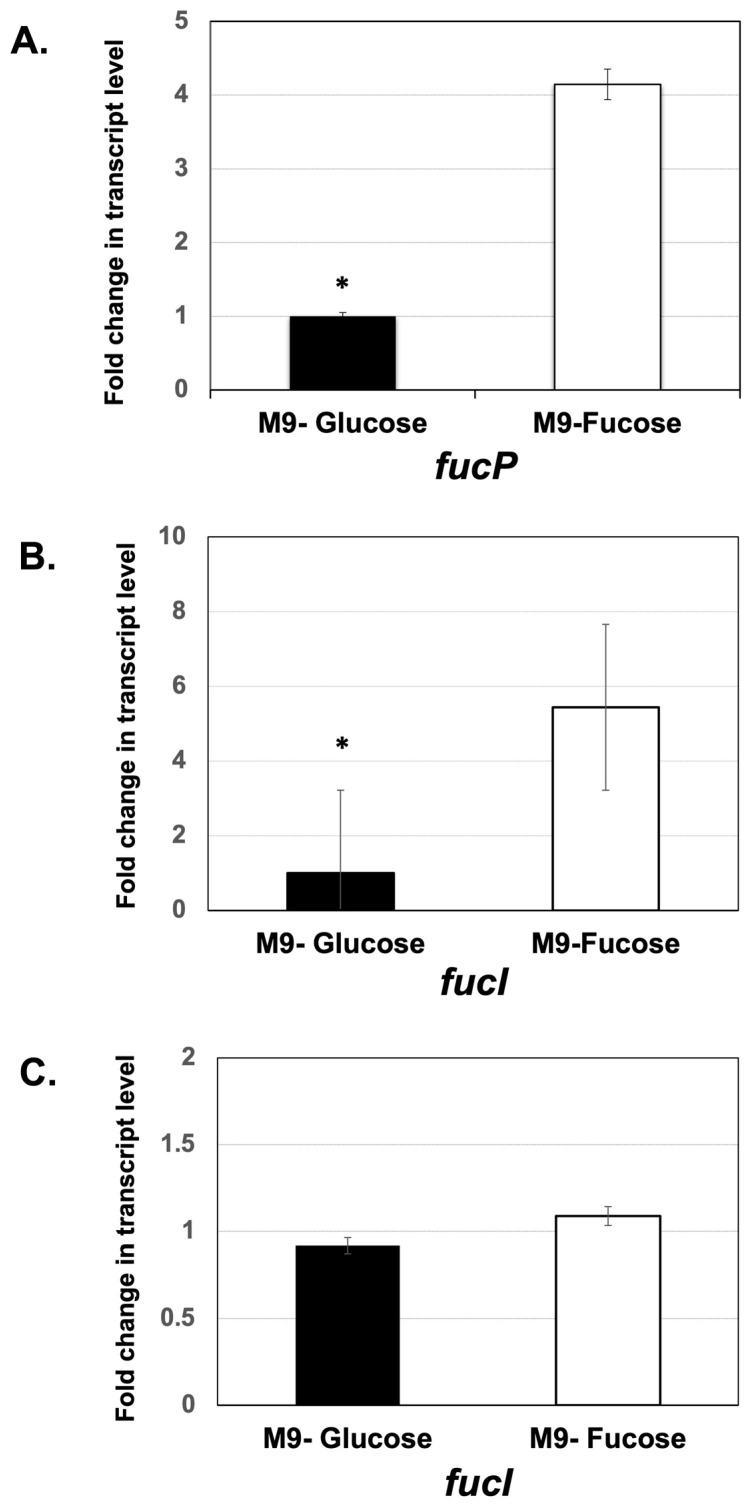
Relative quantification of transcript levels of *fucP* (**A**) and *fucI* (**B**) in *E. coli* K12 when grown in minimal media supplemented with fucose. A similar analysis of *fucI* transcription was performed in *E. coli* BL21 (**C**). Significant differences from the M9-Fucose strain are indicated by an asterisk * (*p*-value < 0.005).

The transcription of *fucP* and *fucI* genes was higher in *E. coli* K12 grown in fucose condition than grown in glucose. The increase was statistically significant for *fucP* in *E. coli* K12 (*p* < 0.005). Conversely, no statistically significant difference was found for the *fucI* gene in *E. coli* K12 (Figure 5). With *E. coli* BL21, which lacks *fucP*, only the *fucI* gene expression was tested, and it was statistically significantly higher as well (Figure 4). Such results are consistent with the induction of the operon by fucose.

## 4. Discussion

The human gut harbors a wide variety of bacteria, making up the gastrointestinal microbiota, which coexists in a mutually beneficial state with its host. In this symbiotic relationship, the microbiota contributes to nutrient degradation and metabolism, acts as a barrier that confers resistance to pathogen colonization, and helps develop and maintain proper immune responses. In return, the host provides the niche and nutrients essential for microbial propagation and population survival. This mutualistic relationship is beneficial for both microbiota and host in many ways [33]. This relationship can sometimes change its nature as the immense number of microbes present in the gut can become threatening to the host. Therefore, the intestinal epithelium is covered with a dense layer of mucus to prevent the translocation of the gut microbiota into underlying tissues [34].

The intestinal microbiota is often described as a ‘functional organ’ as it regulates plentiful physiological functions of the host, such as digestion, metabolism, and immunity. We rather describe it as a dynamic cloud, which varies temporally and spatially [35]. One of the main functions of the gut microbiota is protecting the host GIT against colonization by exogenous pathogens or endogenous opportunists [36]. This protection is achieved either via direct competition for nutrients or through modulation of host immune responses [37]. Changes in the intestinal microbiota composition are associated with a series of diseases and dysfunctions, including inflammatory bowel disease, obesity, colorectal cancer, and type-2 diabetes. The compositional changes in intestinal microbiota also increase the intestinal susceptibility to infection, as the indigenous intestinal microbiota-mediated innate and adaptive defense is disrupted [38].

The GIT is lined by a protective mucus barrier, the key ingredients of which are mucin glycoproteins [2]. One of the best-studied mucin-degrading species is *Bacteroides thetaiotaomicron*, a common member of the intestinal microbiota. Its genome encodes over 80 polysaccharide utilization loci, including many that are known or predicted to be involved in the breakdown and utilization of mucin O-glycans [39].

*E. coli* is one of the abundant commensals present in the intestine of humans and different animal species [40]. Central metabolic pathways in *E. coli* are highly conserved and constitute a significant part of the core *E. coli* genome [7,33]. The various nutritional sources provided by the gut mucosa are considered the fuel and energy on which the bacterial cell thrives. It has the ability to break down the monosaccharides available for its growth but lacks the ability to break down complex polysaccharides, and this is where the *Bacteroides* come for assistance. *E. coli* does not express α-L-fucosidases, so it depends on other bacterial groups for L-fucose release from fucose-containing glycans [33,41].

In this study, evidence is presented for strain-specific utilization of fucose, a prominent monosaccharide component of mucin and other glycoproteins, to support growth. Fucose utilization has been described for both pathogenic and commensal bacteria, including *Salmonella enterica* serovar Typhimurium, *E. coli*, *Klebsiella pneumoniae*, *Roseburia inulinivorans*, and *B. thetaiotaomicron*. Under both anaerobic and aerobic conditions, L-fucose is metabolized to dihydroxyacetone phosphate (DHAP) and L-lactaldehyde by the key enzymes fucose isomerase, fuculose kinase, and fuculose aldolase (FucI, FucK, and FucA, respectively) [18].

Throughout the literature, genes responsible for fucose utilization differed from one species to another; therefore, an important aim of this study was to substantiate the genes present in the fucose operon in key members of the family *Enterobacteriaceae*, especially *E. coli*. An extensive comparative genomic analysis showed key differences in fucose utilization and fucose operon structure in members of the *Enterobacteriaceae* family [42]. Upon the analysis of the different variants of fucose operon in *E. coli*, we found that certain strains contained the entire operon, such as *E. coli* K12 substr. MG1655, while others lacked certain crucial genes. This allowed further investigation of how these strains, lacking the permease-encoding gene, *fucP*, could metabolize the sugar and how they were able to enter the sugar inside the bacterial cell. The investigation delineated another permease (from the ABC transporter family) in the variant strains lacking *fucP*, for example, *E. coli* B strain BL21.

The L-fucose permease, FucP, is a member of the major facilitator superfamily (MFS) transporters belonging to the fucose H^+^ symporter family (FHS). The MFS transporter is responsible for transporting many substances across the cell membrane, such as sugars, organic acids, phosphates, nitrates, lipids, and metals [27,43].

We used PCR to confirm the obtained computational results, which revealed a broad distribution of Variant 1 (normal fucose operon) in 85% of clinical samples collected throughout the study, and the presence of this locus was consistently associated with fucose utilization. However, the remaining 15% of the collected samples lacked the *fucP* gene. Interestingly, all the samples lacking *fucP* contained the ABC transporter permease. This suggests that the ABC transporter may be an alternative to the MFS fucose transporter. However, it seems from our experimental results that FucP is a more efficient transporter, from a fucose utilization perspective. At high glucose concentration (4 g/L), *E. coli* B (BL21) grew faster than *E. coli* K12 (MG1655), unlike when grown at high fucose concentration. This observation led to investigations to identify the metabolic differences between the two strains. After reviewing the literature, we found examples of metabolic variations between different K strains and B strains. For example, when grown at high glucose concentration (20–40 g/L), *E. coli* B (BL21) grew faster, to higher bacterial concentration, and produced less acetate than *E. coli* K (JM 109) [16].

With regards to growth in fucose as a sole carbon source, we showed that wild-type K-12 MG1655 grew slower with a relatively long lag time; yet, BL21 grew even slower in fucose than in glucose, and with more than 12 h of lag time. The slow growth rate for K12 in M9 supplemented with fucose, as opposed to glucose, demonstrates that these sugars are not equivalent in providing carbon and energy to the bacterial cell. The slow growth rate in BL21 is expected, as they lack the *fucP* gene, encoding a fucose transporter, and they use another ABC transporter for fucose inside the cell.

A recent multi-omics study conducted by Kim et al. [44] used metabolomics and transcriptomics to show that the metabolic activity of *E. coli* is different when the bacteria were grown on two different carbon sources, fucose or glucose. The authors noted that culturing *E. coli* on fucose leads to inefficient carbon metabolism, resulting in fuculose accumulation and slow growth [45]. In another instance, when the growth of various *E. coli* strains on L-fucose was compared, *E. coli* B strains could not grow with L-fucose as a sole carbon and energy source, unlike K-12 strains. *E. coli* B strains cannot use L-fucose because of the lack of L-fuculose-1-phosphate aldolase activity, but aldolase-positive revertant variants were experimentally shown to grow on L-fucose as a sole carbon and energy source [44].

Here, growth analysis was also performed on mutant strains obtained from the Keio collection lacking *fucP*, *fucI*, *fucO,* and *fucU*. All mutant strains grew normally on M9 minimal medium supplemented with glucose, as expected; however, their growth in M9 minimal medium supplemented with fucose significantly differed for each mutant strain. The Δ*fucP* strain behaved as BL21 in the fucose M9 minimal medium, which indicates the survival mechanism of the bacterium in choosing another ABC transporter in the absence of the operon’s permease, *fucP*. The Δ*fucI* strain failed to grow on a minimal medium supplemented with 0.4% L-fucose, most likely because the missing fucose isomerase is a key enzyme in breaking down fucose inside the bacterial cell. The Δ*fucU* strain was characterized by an extended lag phase because mutarotase activity is absent. The absence of FucU could leave the cell to the spontaneous α-to-β conversion of a pyranose (six-membered) ring, which is not rapid enough to support fast-energy generation by an organism [46].

The reduced growth rate in Δ*fucP* on L-fucose, compared to the inability Δ*fucI* to grow, suggests the importance of the isomerase enzyme in catabolizing L-fucose. However, when lacking the permease, the bacterium may resort to other less effective transporters to transport the sugar inside the cell, such as the ABC transporter discovered through genomic analysis. A study analyzed the fucose operon in *Lactobacillus rhamnosus* GG using mutant construction reported that upon the growth of the *L. rhamnosus* strain in minimal media supplemented with L-fucose, the *fucI* mutant strain failed to grow and acidify the media. However, the Δ*fucP* strain grew but with a much-reduced growth rate than the wild-type strain [41].

Δ*fucO* shared a similar pattern to the growth of K12 in a minimal medium supplemented with fucose, and this suggests that the mutation affects the byproducts of metabolism and not the sugar utilization itself.

Further assessment of the fucose utilization genes and their importance to *E. coli* was achieved through transcriptional analysis. RT PCR was performed to evaluate the transcription of *fucP* as well as *fucI* (in two different strains containing different fucose operon variants). In *E. coli* K12, *fucP* and *fucI* expression was higher in glucose than in fucose because the fucose supplemented in the media acted as an inducer. In 2015, Vital et al. [47] showed that affinities to certain substrates characteristic of the individual habitats, such as fucose, a common component of host mucus glycans contributed to ecology-specific gene expression profiles [47].

The ability of *E. coli* to deal with constantly changing environmental conditions is highlighted in the meta-analysis of gene expression data from GEO, which shows that the majority of genes were markedly expressed in some culture conditions, with variability, and is consistent with previous observations for *E. coli* and other taxa. These results emphasize the relative nature of comparative gene-expression investigations, as the culture condition enormously influences results for in-between strain comparisons.

The human GIT is normally co-colonized with commensal *E. coli* belonging to five different strains, on average, whereas pathogenic *E. coli* strains are invaders that take over the intestine in the presence of other harmless strains [7]. Thus, metabolic switching might provide means for several commensals to co-inhabit the intestine by dynamic adaptation to host-strain-nutrient variations.

This study represents a first step towards characterizing these nutrient adaptation patterns. However, there are inevitable limitations and unanswered questions that will be addressed in future studies. For example, only two among the model *E. coli* type strains have been experimentally studied (K12 and B) as they represented the two major fucose pathway variants that we identified. However, more strains that are representative of various industrial types and pathotypes can be further studied for dissecting more subgroups of the pathway variants. More isogenic mutants in different strain backgrounds could also improve our understanding of the impact of every step in the fucose utilization pathway on growth and fitness. Finally, future studies will address the distribution of fucose pathway variants in various *E. coli* pathotypes and will explore the impact of these variations on inter-bacterial signaling and virulence in vivo.

## 5. Conclusions

Taken together, our results suggest that there may be a difference of central importance between how *E. coli* BL21 and MG1655 sense and metabolize nutrient choices in the intestine in response to the intestinal environment. This was shown by both phenotypic and genotypic studies. Breaking down the signals that affect *E. coli* nutrient choice in vivo and the mechanisms by which *E. coli* alters its catabolic capacity in response to these signals will likely allow a better understanding of the relationship between gut microbiota and the GI tract.

Our findings join other studies to demonstrate how individual bacteria exhibit different preferences for carbohydrates produced from the host diet and mucosal secretions. This work provides insights into L-fucose catabolism and its regulation in different enterobacterial species, with particular emphasis on *E. coli*. In conclusion, we show how *E. coli*, responds to one of the nutrients in the complex gut ecosystem and open the way for other studies to explore how this may affect the behavior of *E. coli* in the gut community.

## Figures and Tables

**Table 1:** List of primers used in this study.

Primer	Target Gene	Primer Sequence (5′-3′)	Use	Product Length
*fucP*_F	*fucP*	TTCTGAAACGGGCATGAAAT	PCR	1697
*fucP*_R		CCGTAGCTTTCGCCATATTC		
*fucI*_F	*fucI*	ATTATTGGCGGCGGTATTG	PCR	2097
*fucI*_R		CACAGTCGAGTACCAGGATAA		
eco1_F	*eco1*	CGCGAGGAAWATRCCGATAA	PCR	427
eco1_R		CCTGACHGATAAYGCCTTTCT		
*yjfF* _f	*yjfF*	GAGCTCAGCGTRCCATCAAA	PCR	831
*yjfF* _R		CGTCTTTGTGYTGGGYTATCT		
*fucI*B-F	*fucI_B*	CCACTGGACCGATCAATATC	PCR	444
*fucI*B_R		AGGAAGCGGGAAGAGTAA		
*fucP*_F	*fucP_RT*	TYATTGGCGGCGGTATTG	RT- PCR	124
*fucP*_R		CGGAAACGGGCAAAGATAA		
*fucI*_F	*fucI*_RT	CGCCAGCATGAAAGGTAA	RT- PCR	137
*fucI*_R		GACGCAGTTCGGTCATATC		
*ihfB*_F	*IhfB*	GATAGAAAGACTTGCCACCCA	RT- PCR	200
*ihfB*_R		CCAGTTCTACTTTATCGCCAG		

**Table 2:** Variants of fucose operon, based on SEED subsystems analysis.

Variant	Description
1	The entire operon is present (*fucP*, *fucI*, *fucK*, *fucU*, *fucR*, *fucA*, *fucO*)
1.1	The Fuc operon is present + *fucZ*
1.2	A variant of 1 that lacks *fucO* but has *fucZ*
1.3	Fucose operon lacking *fucU*
1.4	Fucose operon lacking *fucO* → no anaerobic production of 1, 2-PDO
2	A variant of the fucose operon that lacks *fucP*, and has an ABC transporter-encoding set of genes, in most cases
2.1	A variant of 2 missing *fucO*
2.2	Fucose operon missing *fucP* without a defined ABC transporter permease
3	Fuc operon + predicted phosphotransferase EIIA and EIIB
4	Fucase + Fuc operon without *fucU* + RR1
4.1	Fucase + Fuc operon lacking *fucP* and no RR1
4.2	Fucase + Fuc operon but lacking *fucO*
4.3	Fucase + Fuc operon
5	Missing *fucR*

## Data Availability

Raw data used for the analysis are provided in Supplementary Table S1. Subsystems data are publicly available at URL: http://pubseed.theseed.org//SubsysEditor.cgi?page=ShowSubsystem&subsystem=L-fucose_utilization_and_AldA_biosynthesis (link last checked on 25 April 2023). All experimental results, gel pictures, and glycerol stocks of bacterial isolates are available upon request.

## Supplementary Materials

The following supporting information can be downloaded at: https://www.mdpi.com/article/10.3390/microorganisms11051265/s1, Figure S1: Sources of collected isolates; Table S1: Raw data and metadata of 483 analyzed samples from 52 GEO data sets; Table S2: Post hoc pairwise comparisons of data of growth curves (Figure 2).
