# Supplementary material for: In Silico and In Vitro Investigation of the Distribution and Expression of Key Genes in the Fucose Operon of Escherichia coli
Source: Microorganisms. 2023 May 11;11(5):1265. doi: 10.3390/microorganisms11051265 (PMC10221146; doi:10.3390/microorganisms11051265)
Supplement: Supplementary file 1 [file microorganisms-11-01265-s001.zip › TableS2_ANOVA.pdf]

Table S2: Two-way ANOVA analysis of Growth Curves

Results of analysis by GraphPad Prism®, Version 6, (GraphPad Software , San Diego, CA, USA)

Bonferroni posttests

K12-GLucose vs BL21-Glucose

Time	K12-GLucose	BL21-Glucose	Difference	95% CI of diff.
0	0.01	0.01	0	-0.1924 to 0.1924
4	0.269	0.434	0.165	-0.02739 to 0.3574
6	0.7767	0.998	0.2213	0.02894 to 0.4137
8	1.08	1.458	0.378	0.1856 to 0.5704
10	1.366	1.753	0.3867	0.1943 to 0.5791
24	1.759	1.85	0.09067	-0.1017 to 0.2831
48	1.877	1.95	0.07367	-0.1187 to 0.2661

Time	Difference	t	P value	Summary
0	0	0	P > 0.05	ns
4	0.165	2.981	P < 0.05	*
6	0.2213	3.998	P<0.001	***
8	0.378	6.828	P<0.001	***
10	0.3867	6.985	P<0.001	***
24	0.09067	1.638	P > 0.05	ns
48	0.07367	1.331	P > 0.05	ns

K12-GLucose vs ΔP-Glucose

Time	K12-GLucose	ΔP-Glucose	Difference	95% CI of diff.
0	0.01	0.01	0	-0.1924 to 0.1924
4	0.269	0.2123	-0.05667	-0.2491 to 0.1357
6	0.7767	0.4333	-0.3433	-0.5357 to -0.1509
8	1.08	0.773	-0.307	-0.4994 to -0.1146
10	1.366	1.098	-0.2683	-0.4607 to -0.07594
24	1.759	1.52	-0.2393	-0.4317 to -0.04694
48	1.877	1.766	-0.1103	-0.3027 to 0.08206

Time	Difference	t	P value	Summary
0	0	0	P > 0.05	ns
4	-0.05667	1.024	P > 0.05	ns
6	-0.3433	6.202	P<0.001	***
8	-0.307	5.546	P<0.001	***
10	-0.2683	4.847	P<0.001	***
24	-0.2393	4.323	P<0.001	***
48	-0.1103	1.993	P > 0.05	ns

K12-GLucose vs ΔI-Glucose

Time	K12-GLucose	ΔI-Glucose	Difference	95% CI of diff.
0	0.01	0.01	0	-0.1924 to 0.1924
4	0.269	0.294	0.025	-0.1674 to 0.2174
6	0.7767	0.7043	-0.07233	-0.2647 to 0.1201
8	1.08	1.004	-0.07633	-0.2687 to 0.1161
10	1.366	1.395	0.02867	-0.1637 to 0.2211
24	1.759	1.733	-0.02667	-0.2191 to 0.1657
48	1.877	1.861	-0.01533	-0.2077 to 0.1771

Time	Difference	t	P value	Summary
0	0	0	P > 0.05	ns
4	0.025	0.4516	P > 0.05	ns
6	-0.07233	1.307	P > 0.05	ns
8	-0.07633	1.379	P > 0.05	ns
10	0.02867	0.5178	P > 0.05	ns
24	-0.02667	0.4817	P > 0.05	ns
48	-0.01533	0.277	P > 0.05	ns

K12-GLucose vs ΔU-Glucose

Time	K12-GLucose	ΔU-Glucose	Difference	95% CI of diff.
0	0.01	0.01	0	-0.1924 to 0.1924
4	0.269	0.2633	-0.005667	-0.1981 to 0.1867
6	0.7767	0.6133	-0.1633	-0.3557 to 0.02906
8	1.08	1.038	-0.042	-0.2344 to 0.1504
10	1.366	1.294	-0.072	-0.2644 to 0.1204
24	1.759	1.569	-0.19	-0.3824 to 0.002391
48	1.877	1.82	-0.05667	-0.2491 to 0.1357

Time	Difference	t	P value	Summary
0	0	0	P > 0.05	ns
4	-0.005667	0.1024	P > 0.05	ns
6	-0.1633	2.95	P < 0.05	*
8	-0.042	0.7587	P > 0.05	ns
10	-0.072	1.301	P > 0.05	ns
24	-0.19	3.432	P<0.01	**
48	-0.05667	1.024	P > 0.05	ns

K12-GLucose vs ΔO-Glucose

Time	K12-GLucose	ΔO-Glucose	Difference	95% CI of diff.
0	0.01	0.01	0	-0.1924 to 0.1924
4	0.269	0.2253	-0.04367	-0.2361 to 0.1487
6	0.7767	0.548	-0.2287	-0.4211 to -0.03628
8	1.08	0.7667	-0.3133	-0.5057 to -0.1209
10	1.366	1.173	-0.1927	-0.3851 to -0.0002764
24	1.759	1.531	-0.2283	-0.4207 to -0.03594
48	1.877	1.75	-0.1263	-0.3187 to 0.06606

Time	Difference	t	P value	Summary
0	0	0	P > 0.05	ns
4	-0.04367	0.7888	P > 0.05	ns
6	-0.2287	4.131	P<0.001	***
8	-0.3133	5.66	P<0.001	***
10	-0.1927	3.48	P<0.01	**
24	-0.2283	4.125	P<0.001	***
48	-0.1263	2.282	P > 0.05	ns

K12-GLucose vs K12-Fucose

Time	K12-GLucose	K12-Fucose	Difference	95% CI of diff.
0	0.01	0.01	0	-0.1924 to 0.1924
4	0.269	0.1247	-0.1443	-0.3367 to 0.04806
6	0.7767	0.2753	-0.5013	-0.6937 to -0.3089
8	1.08	0.3403	-0.7397	-0.9321 to -0.5473
10	1.366	0.6667	-0.6993	-0.8917 to -0.5069
24	1.759	1.573	-0.186	-0.3784 to 0.006390
48	1.877	1.821	-0.05567	-0.2481 to 0.1367

Time	Difference	t	P value	Summary
0	0	0	P > 0.05	ns
4	-0.1443	2.607	P > 0.05	ns
6	-0.5013	9.056	P<0.001	***
8	-0.7397	13.36	P<0.001	***
10	-0.6993	12.63	P<0.001	***
24	-0.186	3.36	P<0.01	**
48	-0.05567	1.006	P > 0.05	ns

K12-GLucose vs BL21-Fucose

Time	K12-GLucose	BL21-Fucose	Difference	95% CI of diff.
0	0.01	0.01	0	-0.1924 to 0.1924
4	0.269	0.01633	-0.2527	-0.4451 to -0.06028
6	0.7767	0.04967	-0.727	-0.9194 to -0.5346
8	1.08	0.02167	-1.058	-1.251 to -0.8659
10	1.366	0.055	-1.311	-1.503 to -1.119
24	1.759	0.1973	-1.562	-1.754 to -1.370
48	1.877	1.267	-0.61	-0.8024 to -0.4176

Time	Difference	t	P value	Summary
0	0	0	P > 0.05	ns
4	-0.2527	4.564	P<0.001	***
6	-0.727	13.13	P<0.001	***
8	-1.058	19.12	P<0.001	***
10	-1.311	23.68	P<0.001	***
24	-1.562	28.22	P<0.001	***
48	-0.61	11.02	P<0.001	***

K12-GLucose vs ΔP-Fucose

Time	K12-GLucose	ΔP-Fucose	Difference	95% CI of diff.
0	0.01	0.01	0	-0.1924 to 0.1924
4	0.269	0.01467	-0.2543	-0.4467 to -0.06194
6	0.7767	0.02767	-0.749	-0.9414 to -0.5566
8	1.08	0.03467	-1.045	-1.238 to -0.8529
10	1.366	0.016	-1.35	-1.542 to -1.158
24	1.759	0.03933	-1.72	-1.912 to -1.528
48	1.877	0.712	-1.165	-1.357 to -0.9723

Time	Difference	t	P value	Summary
0	0	0	P > 0.05	ns
4	-0.2543	4.594	P<0.001	***
6	-0.749	13.53	P<0.001	***
8	-1.045	18.88	P<0.001	***
10	-1.35	24.39	P<0.001	***
24	-1.72	31.07	P<0.001	***
48	-1.165	21.04	P<0.001	***

K12-GLucose vs ΔI-Fucose

Time	K12-GLucose	ΔI-Fucose	Difference	95% CI of diff.
0	0.01	0.01	0	-0.1924 to 0.1924
4	0.269	0.021	-0.248	-0.4404 to -0.05561
6	0.7767	0.02567	-0.751	-0.9434 to -0.5586
8	1.08	0.03233	-1.048	-1.240 to -0.8553
10	1.366	0.013	-1.353	-1.545 to -1.161
24	1.759	0.033	-1.726	-1.919 to -1.534
48	1.877	0.055	-1.822	-2.014 to -1.629

Time	Difference	t	P value	Summary
0	0	0	P > 0.05	ns
4	-0.248	4.48	P<0.001	***
6	-0.751	13.57	P<0.001	***
8	-1.048	18.92	P<0.001	***
10	-1.353	24.44	P<0.001	***
24	-1.726	31.18	P<0.001	***
48	-1.822	32.91	P<0.001	***

K12-GLucose vs ΔU-Fucose

Time	K12-GLucose	ΔU-Fucose	Difference	95% CI of diff.
0	0.01	0.01	0	-0.1924 to 0.1924
4	0.269	0.03433	-0.2347	-0.4271 to -0.04228
6	0.7767	0.07467	-0.702	-0.8944 to -0.5096
8	1.08	0.02167	-1.058	-1.251 to -0.8659
10	1.366	0.004	-1.362	-1.554 to -1.170
24	1.759	0.063	-1.696	-1.889 to -1.504
48	1.877	0.6817	-1.195	-1.387 to -1.003

Time	Difference	t	P value	Summary
0	0	0	P > 0.05	ns
4	-0.2347	4.239	P<0.001	***
6	-0.702	12.68	P<0.001	***
8	-1.058	19.12	P<0.001	***
10	-1.362	24.6	P<0.001	***
24	-1.696	30.64	P<0.001	***
48	-1.195	21.59	P<0.001	***

K12-GLucose vs ΔO-Fucose

Time	K12-GLucose	ΔO-Fucose	Difference	95% CI of diff.
0	0.01	0.01	0	-0.1924 to 0.1924
4	0.269	0.1223	-0.1467	-0.3391 to 0.04572
6	0.7767	0.1463	-0.6303	-0.8227 to -0.4379
8	1.08	0.1527	-0.9273	-1.120 to -0.7349
10	1.366	0.2593	-1.107	-1.299 to -0.9143
24	1.759	0.856	-0.9033	-1.096 to -0.7109
48	1.877	1.283	-0.5933	-0.7857 to -0.4009

Time	Difference	t	P value	Summary
0	0	0	P > 0.05	ns
4	-0.1467	2.649	P > 0.05	ns
6	-0.6303	11.39	P<0.001	***
8	-0.9273	16.75	P<0.001	***
10	-1.107	19.99	P<0.001	***
24	-0.9033	16.32	P<0.001	***
48	-0.5933	10.72	P<0.001	***

Two-way ANOVA results

Source of Variation	% of total variation	P value
Interaction	17.15	< 0.0001
Column Factor	35.47	< 0.0001
Time	46.69	< 0.0001

Source of Variation	P value summary	Significant?
Interaction	***	Yes
Column Factor	***	Yes
Time	***	Yes

Source of Variation	Df	Sum-of-squares	Mean square	F
Interaction	66	19.3	0.2924	63.6
Column Factor	11	39.9	3.627	789.1
Time	6	52.53	8.754	1904
Residual	168	0.7723	0.004597	

# Missing values	0
