# Supplementary figures and images for: In Silico and In Vitro Investigation of the Distribution and Expression of Key Genes in the Fucose Operon of Escherichia coli
Source: Microorganisms. 2023 May 11;11(5):1265. doi: 10.3390/microorganisms11051265 (PMC10221146; doi:10.3390/microorganisms11051265)

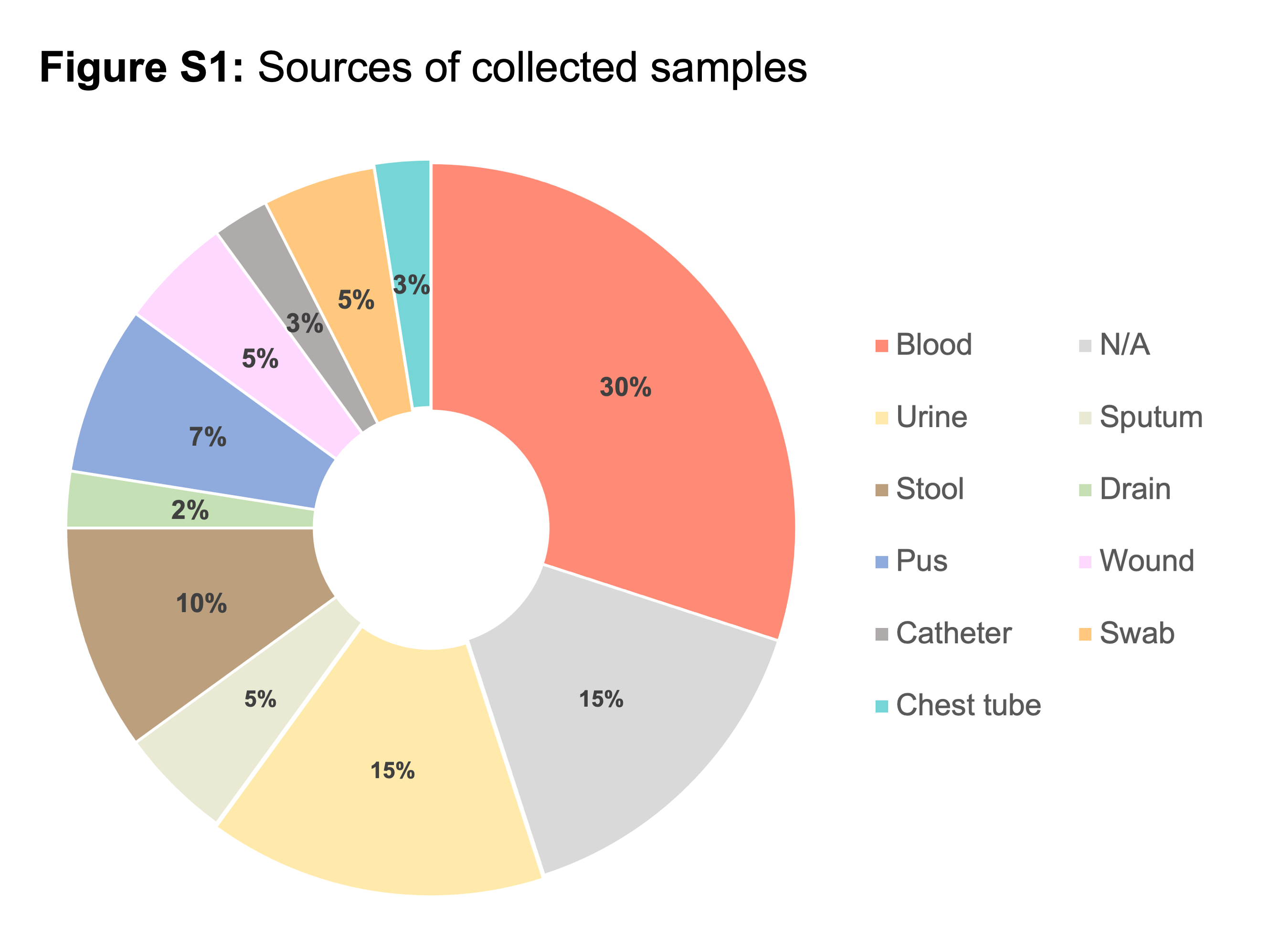

Supplement: Supplementary file 1 [file microorganisms-11-01265-s001.zip › FigS1.png]
